# Respiratory Viral and Bacterial Exacerbations of COPD—The Role of the Airway Epithelium

**DOI:** 10.3390/cells11091416

**Published:** 2022-04-22

**Authors:** Michelle E. Love, David Proud

**Affiliations:** Department of Physiology & Pharmacology, Snyder Institute for Chronic Diseases, Cumming School of Medicine, University of Calgary, Calgary, AB T2N 4Z6, Canada; melove@ucalgary.ca

**Keywords:** airway epithelial cells, rhinovirus, bacteria, innate immunity, host defense, inflammation

## Abstract

COPD is a leading cause of death worldwide, with acute exacerbations being a major contributor to disease morbidity and mortality. Indeed, exacerbations are associated with loss of lung function, and exacerbation frequency predicts poor prognosis. Respiratory infections are important triggers of acute exacerbations of COPD. This review examines the role of bacterial and viral infections, along with co-infections, in the pathogenesis of COPD exacerbations. Because the airway epithelium is the initial site of exposure both to cigarette smoke (or other pollutants) and to inhaled pathogens, we will focus on the role of airway epithelial cell responses in regulating the pathophysiology of exacerbations of COPD. This will include an examination of the interactions of cigarette smoke alone, and in combination with viral and bacterial exposures in modulating epithelial function and inflammatory and host defense pathways in the airways during COPD. Finally, we will briefly examine current and potential medication approaches to treat acute exacerbations of COPD triggered by respiratory infections.

## 1. Introduction

Chronic obstructive pulmonary disease (COPD) is a multifaceted inflammatory disease of the lungs that is now one of the top three causes of death worldwide, with most deaths occurring in low- and middle-income countries [1,2]. The disease is characterized by chronic inflammation of the airways accompanied by persistent airflow limitation and dyspnea, with mucous hyper secretion, chronic cough and recurrent lower respiratory infections also being common features. While biomass smoke, environmental pollutants, occupational exposures and genetic factors can lead to the development of COPD, cigarette smoke is the leading risk factor in the developed world [1]. COPD manifests as periods of stable chronic symptoms interrupted by periods of acute exacerbations, defined as periods of worsening of symptoms requiring increased medication or hospitalization. Even a single COPD exacerbation can result in a significant increase in the rate of decline in lung function [3]. However, the severity and frequency of COPD exacerbations increases with disease progression [4], and frequent exacerbations are associated with a more rapid decline in lung function, poorer quality of life and increased mortality [5,6,7]. Patients can experience a range of symptoms during exacerbation but the most characteristic are increased dyspnea, increased sputum volume and increased sputum purulence [8]. In addition to their impact on disease outcomes, acute exacerbations of COPD leading to hospitalizations are major drivers of healthcare costs accounting for 50–80% of direct medical costs [9,10].

At the onset of exacerbations 35% of patients report cold-like symptoms [11], and it is clear that exacerbations of COPD are frequently associated with viral and/or bacterial infections [12,13,14,15]. Although the percentage varies in individual studies, it appears that approximately 20–30% of patients with acute exacerbations of COPD have a detectable bacterial infection, 20–50% have a viral infection and approximately 25% have a bacterial viral co-infection [16,17]. The primary viral pathogen detected in patients with exacerbation of COPD is rhinovirus [14], while *Haemophilius influenzae* is the most commonly observed bacterial pathogen [17,18]. The airway epithelium is the initial site of interactions of pathogens with the airway and is also the primary site of viral replication. As such, it is important to briefly consider the properties of airway epithelial cells that contribute to the maintenance of airway homeostasis and the response to pathogens.

In the current article we will review the role of the epithelium in host defense and consider how the properties of the epithelium are modified by cigarette smoke, as well as by viral and bacterial infections. Co-infections will also be reviewed as will the current status of therapies for exacerbations of COPD. In this review we focus on general, broadly applicable concepts and do not focus on specific subgroups of patients. We also focus on common seasonal viruses and do not consider COVID-19 for several reasons. The relationship of the COVID-19 epidemic with exacerbations of COPD is complex and still evolving. Moreover, distinguishing the symptoms of a typical exacerbation from COVID-19 infection is difficult, and it is unclear whether management should target COVID-19 with antiviral agents or focus on typical management of COVID-19 exacerbations.

## 2. Role of the Epithelium in Host Defense

A major function of the airway epithelium is to serve as a barrier to inhaled pathogens. The first line of this defense in the larger airways is mucociliary clearance, by which pathogens are trapped in a viscous surface layer and removed before they can act with surface-binding sites on airway epithelial cells. Mucociliary clearance involves coordinated beating of cilia through a watery periciliary layer to transport particulates and pathogens trapped in the upper mucus gel layer towards the trachea. The mucus layer is comprised of a mixture of aqueous fluid and high-molecular-weight mucin glycoproteins which contain binding sites for pathogens. In humans, MUC5AC and MUC5B are the dominant secreted gel-forming mucins [19,20], while MUC2 is only a minor constituent of the mucous layer [20]. By contrast, MUC1, MUC4 and MUC16, the dominant tethered mucins, remain attached to the epithelial surface, serving as an added layer of protection against penetration by noxious agents [20]. The structural diversity of mucin glycoproteins means they contain carbohydrate moieties that recognize adhesins and hemagglutinins from numerous pathogens including *Haemophilius influenzae*, *Pseudomonas aeruginosa*, *Staphylococcus aureus*, *Streptococcus pneumoniae*, and influenza virus [21]. If pathogens penetrate the mucociliary layer, the airway epithelium forms a pseudostratified physical barrier to segregate the luminal contents of the airway from the lamina propria. A number of intercellular junctional complexes including tight junctions, adherens junctions, gap junctions, and desmosomes collectively contribute to maintaining cell polarity and a tight barrier. Epithelial barrier function is primarily controlled by the expression and organization of tight junctions, which are apically expressed intercellular adhesion complexes that selectively regulate the passage of macromolecules, water, and ions [22]. Tight junctions are comprised of three main types of transmembrane proteins: claudins, occludin, and junctional adhesion molecules (JAMs), which are connected to the actin cytoskeleton via junctional plaques that include members of the zonula occludens protein family, which help regulate junctional assembly [23].

In addition to a physical barrier function, epithelial cells express a range of pattern recognition molecules, including Toll-like receptors, NOD-like receptors and mannose-binding lectin, that recognize pathogens and subsequently initiate proinflammatory responses, including the release of chemokines to recruit phagocytes and other cells, as well as host defense responses that include the production of a wide array of antibacterial and antiviral molecules [24]. Peptides and proteins with direct antimicrobial activity include lysozyme, lactoferrin, secretory leukocyte protease inhibitor, members of the β-defensin family of peptides, cathelicidin and surfactant proteins [24]. These peptides and proteins are complemented by proteases and antiproteases, and by reactive oxygen species (ROS) and reactive nitrogen species (RNS).

Although macrophages and leukocytes are usually considered to be the major sources of ROS and RNS in the lung, airway epithelial cells express NADPH oxidase, xanthine oxidase and dual oxidases (Duox) 1 and 2, all of which can contribute to ROS production. For example, epithelial cells generate superoxide in response to multiple stimuli, including mechanical strain [25]. In addition, epithelial cells generate hydrogen peroxide mainly via the activity of Duox enzymes. Levels of hydrogen peroxide produced at the airway surface from normal epithelial cells are sufficient to support the generation of bacteriocidal hypothiocyanate, a pathway that is inhibited in cystic fibrosis [26]. The generation of ROS can modify epithelial function by activating transcription factors to increase expression of cytokines, chemokines and adhesion molecules. To help mitigate the effects of excessive ROS generation in the airways, epithelial cells also express several antioxidant pathways. The potential of cigarette smoke to disrupt oxidant–antioxidant balance may be a contributing factor to the pathogenesis of COPD.

The major RNS generated by the epithelium is nitric oxide (NO). This is generated from L-arginine, predominantly by the inducible, type 2 isoform (iNOS) of nitric oxide synthase (NOS), which is the major form expressed in the epithelium. It was initially suggested that NO was proinflammatory in the airways because it induces mucus secretion and prostanoid production, modulates ion transport, triggers vasodilation and, under some conditions, enhances vascular permeability. Moreover, it can induce tissue damage via generation of peroxynitrite [27]. On the other hand, NO is bronchodilatory and increases ciliary beat frequency, inhibits vascular permeability at inflammatory sites and inhibits adhesion processes involved in inflammatory cell recruitment [27,28]. NO can also exert antimicrobial and antiviral effects and has been shown to inhibit replication of human rhinovirus [29]. Thus, its role in disease processes is likely complex. There is evidence, however, for increased expression of iNOS and increased nitrositive stress in patients with COPD [30,31].

## 3. Cigarette Smoke Impairs Mucociliary Clearance and Barrier Function

**Figure 1:**
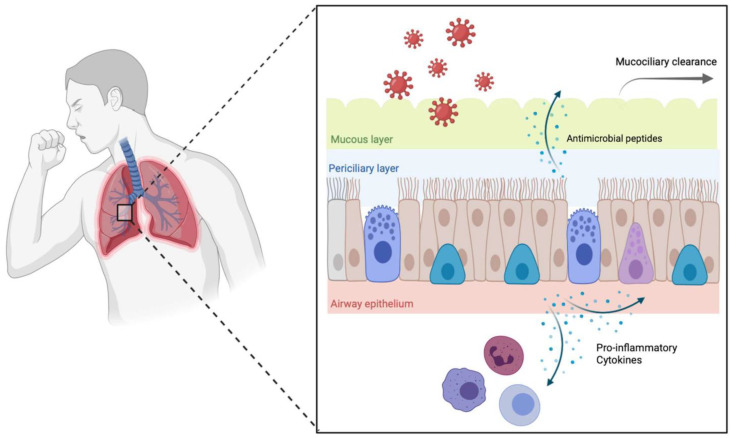
Barrier function, mucociliary clearance, and host defenses in the normal airway epithelium.

Although the airway epithelium displays several lines of defense to inhaled pathogens (Figure 1), cigarette smoke can modify many of these epithelial functions. Cigarette smoke-induced injury to the airway epithelium promotes epithelial remodeling and pro-inflammatory signaling, resulting in impaired mucociliary clearance and disrupted barrier function. This creates an environment that is more favorable for microbial growth and persistence in the lungs.

Cigarette smoke exposure causes several histological changes in the airway epithelium and associated structures including submucosal gland hypertrophy, goblet cell hyperplasia, reduced club cell numbers and increased inflammatory cell infiltrates [32,33,34,35]. In differentiated air–liquid interface cultures, exposure to cigarette smoke is associated with increased MUC5AC and reduced SCGB1A1 (a club cell marker) positive cells [36,37]. Consistent with these observations, bronchial sections of COPD patients show increased MUC5AC staining in the submucosal glands and epithelial surface compared to healthy controls [38]; moreover, the chronic mucus hypersecretion seen in COPD is associated with increased airflow limitation (FEV_1_) and risk of hospitalization [39]. Concentrations of MUC5AC have been reported to be a marker of chronic bronchitis [40]. In addition to hypersecretion of mucus and MUC proteins, sputum samples obtained from smokers with normal lung function and COPD patients have a higher viscosity (lower hydration) and percentage of solids compared to healthy controls, leading to impaired mucociliary transport [35]. Cigarette smoke reduces the hydration of airway secretions and alters the viscoelastic properties of mucus by altering the function of ion channels important for fluid balance. Kreindler et al. observed reduced chloride secretion in airway cells exposed to cigarette smoke extract [41], which is consistent with reports that the expression and function of the cystic fibrosis transmembrane conductance regulator (CFTR) are reduced upon cigarette smoke exposure [42]. In addition to the effects of cigarette smoke on ion channels, it has also been reported that nicotine can increase mucus viscosity by inhibiting the postexocytotic hydration of released mucins, likely via electrostatic and hydrophobic interactions [43].

In addition to the effects of cigarette smoking on mucus release and viscosity, smoking also has profound effects on ciliary function. Smoking decreases ciliogenesis [44], and reduces the number of ciliated cells [45,46], an effect that can be inhibited by use of an EGFR tyrosine kinase inhibitor [47]. Smoking is also associated with a shortening of airway cilia length, a phenomenon linked to smoking-induced downregulation of genes associated with intraflagellar transport, a key process in producing cilia of normal length [48,49]. Finally, smoking also reduces ciliary beat frequency [45,50,51]. It would obviously be expected that the combined effects of cigarette smoking on ciliogenesis, ciliary length, ciliary beat frequency, and mucus dehydration would lead to impaired mucociliary clearance. In confirmation of this, a study examining saccharin transit time in the nasal epithelium as a measure of mucociliary clearance found a significant reduction in mucociliary clearance in current smokers with or without COPD compared to patients with COPD who were ex-smokers or to healthy subjects [52]. Excess mucus production and impaired mucociliary clearance would be expected to contribute to the formation of mucus plugs in COPD. Subsequent infection with bacterial and viral pathogens could promote further mucus secretion and inflammatory signaling in the airways that may lead to additional airflow limitation and exacerbations in the COPD airways.

As noted above, tight junctions in the airway epithelium play an important role in forming cell–cell contacts and maintaining a physical barrier to pathogens. Cigarette smoke exposure is associated with a reduction in barrier function and changes in junctional protein expression. In agreement with older animal studies where exposure to cigarette smoke led to increased levels of inhaled fluorescein isothiocyanate-dextran appearing in the bloodstream [53], Tatsuta and colleagues demonstrated a dose-dependent increase in the permeability of Calu-3 cell cultures with cigarette smoke extract [54]. Moreover, microarray analysis of bronchial brushings from smokers revealed a global downregulation of genes required for the maintenance of apical junctional complexes [55]. Consistent with this observation, increased permeability of epithelial cultures treated with cigarette smoke is associated with alteration of expression of genes linked to tight junction and adherens junction function [54,56,57,58]. Such losses in barrier function would be expected to enhance the ability of pathogens to penetrate the epithelial layer and may also allow increased passage of inflammatory mediators and fluid.

## 4. Bacteria and COPD Exacerbations

Although the traditional dogma based on standard culture techniques was that the lungs of healthy individuals are sterile, the advent of more sensitive molecular diagnostic techniques has shown this not to be the case. While the bacterial burden is far less than in the gastrointestinal tract, the human respiratory tract nonetheless has a distinct endogenous bacterial profile (microbiome) that can be altered during disease [59]. In healthy individuals, the human lung microbiome is composed primarily of bacteria of the phyla *Bacteroides* and *Firmicutes* [59], with *Prevotella, Veillonella* and *Streptococcus* representing the predominant genera present. Unlike the gastrointestinal tract, which demonstrates considerable spatial variation in microbial diversity, samples taken from the upper and lower lobes are relatively consistent in microbial community composition [59,60]. However, not surprisingly, taxonomic diversity decreases with increased distance from the oral cavity [59].

Although it may seem intuitively obvious that cigarette smoking would alter the respiratory tract microbiome, the existing data do not fully support this concept. A study examining bronchoalveolar lavages from healthy non-smokers and smokers found no significant differences in the respiratory microbiome of the two groups [61]. Opron and colleagues compared healthy never-smokers to smokers with normal lung function and to patients with either mild-to-moderate COPD or with severe COPD [62]. They were unable to identify any microbiota variations that specifically associated with either current smoking or with COPD. Rather they found that differences in overall bacterial composition in the lung were more strongly linked to measures of airway dysfunction rather than to a diagnosis of COPD. These data may indicate that secondary factors in addition to cigarette smoking are needed before microbiome changes are seen. It may also be that disease severity and associated inflammatory and structural changes in the lung are required to support microbiome changes, as several studies have found microbiome changes in severe COPD compared to controls [63,64,65]. In general, in patients with severe COPD, the microbiome shows lower diversity, with a loss of part of the resident flora and an increase in pathogenic bacterial species [64,65]. Importantly, these changes in the microbiome were associated with changes in expression of multiple host genes [65].

Patients with COPD are frequently colonized with pathogenic bacterial species even when their disease is stable [66,67,68,69]. In several studies, commonly detected bacteria include *Haemophilus influenzae*, *Streptococcus pneumoniae*, *Moraxella catarrhalis*, and *Pseudomonas aeruginosa*. The burden of such pathogenic bacteria is often associated with increased markers of airway inflammation [67,68,69]. Indeed, Singh and colleagues demonstrated a relationship between bacterial colonization and bacterial load with sputum inflammatory markers. Levels of CXCL8, interleukin 1β and myeloperoxidase were found to be elevated in COPD patients with lower airway bacterial colonization, and total bacterial load was associated with increasing airway inflammation [70]. There is controversy, however, as to whether dysbiosis of the microbiota in stable disease is predictive of exacerbation frequency. Patel and colleagues reported an increased exacerbation frequency associated with colonization by pathogenic bacteria [68], while other studies have found no relationship between baseline microbial patterns and exacerbation frequency [69], or have found that a subset of exacerbations may be associated with baseline microbial dysbiosis [71]. Longitudinal studies have shown that there appears to be a relationship between temporal variations in bacterial profiles and exacerbation frequency [71,72].

There is considerable overlap between the pathogenic bacterial species observed during exacerbations of COPD and those seen during stable disease. Thus, in patients hospitalized with acute exacerbations of COPD, *Haemophilus influenzae* followed by *Pseudomonas aeruginosa*, *Moroxella catarrhalis*, *Streptococcus pneumoniae* and *Staphylycoccus aureus* are commonly detected [17,73]. This led to the suggestion that exacerbations may be triggered by increases in the load of chronically colonizing bacteria, but a subsequent study demonstrated that concentrations of pre-existing bacterial strains were not higher during exacerbations [74]. Thus, it has now been established that acquisition of new virulent strains of pathogenic bacteria is strongly associated with occurrence of exacerbations [75,76,77,78]. Indeed, is has been shown that, after depletion of commensal bacteria with moxifloxacin treatment, only acquisition of a new pathogenic bacteria was associated with occurrence of an exacerbation [79].

Both pathogen virulence and interactions with the host immune system help to determine the outcome of the acquisition of a new bacterial strain. It has been shown that genomic differences between strains of nontypeable *Haemophilus influenzae* partially account for the outcome of an infection with this organism [80]. In addition, strains of *Haemophilus influenzae* that induce exacerbations show increased adherence to epithelial cells and induce more inflammation, such as production of CXCL8 and recruitment of neutrophils, when compared to chronic colonizing strains [78]. Increases in neutrophils, and in chemokines that recruit and activate these cells are strongly linked to acute exacerbations of COPD [81,82]. Cigarette smoking may be expected to enhance neutrophil recruitment as smoking has also been linked to epithelial production of chemokines, such as CXCL8, that can recruit neutrophils [83,84,85]. These effects may also be exaggerated by the ability of smoking to suppress some aspects of host defense [86].

Antibiotic studies provide further support for the role of bacteria in COPD exacerbations. A systematic review concluded that antibiotics are beneficial in the treatment of exacerbations, particularly in those subjects who produce purulent sputum [87]. The use of prophylactic antibiotics to prevent exacerbations is more controversial. It has been reported that some antibiotics, particularly macrolides, were associated with a reduction in exacerbations [88]. Macrolides have attracted particular attention as they are known to have antiinflammatory and immunomodulatory functions in addition to being antibacterial [89]. Studies using one-year treatment of exacerbation-prone patients with either erythromycin or azithromycin reduced the risk of exacerbations relative to usual care [90,91]. There are no studies, however, going beyond a one-year treatment period, and the benefits seen need to be balanced against the development of microbial resistance and side effects.

The factors that underlie bacteria-associated exacerbations of COPD remain incompletely understood. It has recently been shown that glucose levels are increased in sputum samples from patients with COPD relative to healthy subjects [92]. This enriched glucose environment, along with the increased mucus production and reduced mucociliary clearance seen in COPD patients, would be expected to favor bacterial growth and may have the potential to alter the balance of the microbiome in the lungs [59,92]. In support of this concept, *Pseudomonas aeruginosa* inoculated into sputum obtained from COPD patients was associated with increased growth compared to sputum obtained from controls [92]. It is also likely that cigarette smoking decreases innate lung immune defenses to permit new bacterial infection of the lower airways. This could trigger airway inflammation and further changes to innate immune defense, causing exacerbation of disease and permitting chronic infection. This continuous cycle of infection driving chronic inflammation, acute exacerbations and ultimately progression of the disease has been referred to as the “vicious circle” hypothesis [73].

## 5. Viruses and COPD Exacerbations

Although bacterial infections have long been associated with COPD exacerbations, it was not until the beginning of this century that the use of molecular diagnostics revealed the association of respiratory viruses with exacerbations of COPD. In 2000, Seemungal and colleagues examined 33 patients with COPD both when stable and during subsequent exacerbations. In this small study, ten of 43 exacerbations were associated with rhinovirus infections [93]. Since that time, numerous studies have confirmed that multiple viruses, including rhinovirus, influenza, parainfluenza, coronavirus, and respiratory syncytial virus (RSV), can be detected during exacerbations of COPD [12,13,14,94,95,96]. In virtually all studies, human rhinoviruses were the predominant virus type associated with exacerbations accounting for approximately 40–60% of viruses detected, depending on the study [14,16,95,96]. This likely reflects the relative prevalence of rhinoviruses in circulation, rather than any unusual pathogenicity of rhinoviruses. Nonetheless, the predominance of rhinovirus during exacerbations has made it a common choice for further study.

While detection of viruses during acute exacerbations of COPD confirms an association, it does not establish a causal role in the pathogenesis of exacerbations. To further examine the potential causal relationship between rhinovirus infection and COPD exacerbations, Mallia and colleagues performed experimental rhinovirus infections in patients with mild to moderate COPD [97]. Consistent with a role for viruses in exacerbations, they observed that subjects with COPD developed lower airway symptoms accompanied by airflow obstruction. These subjects also showed increased airway inflammation compared to healthy control subjects [97]. The COPD patients infected with rhinovirus also had an increased viral load, suggesting altered viral handling in COPD. The mechanisms by which rhinovirus can induce exacerbation of COPD, however, are not well understood.

The airway epithelium is the primary site of respiratory viral infection and replication. Such infections lead to a modulation of epithelial phenotype, which includes the induction of a wide range of proinflammatory and host response molecules [98]. Production of type I and type III interferons (IFN), and the subsequent induction of multiple interferon-stimulated genes (ISGs) by the airway epithelium, plays a critical role in defense against respiratory viruses [99]. Mallia and colleagues reported that bronchial alveolar lavage cells obtained from COPD patients and infected ex vivo with rhinovirus showed reduced levels of type I and type III IFNs compared to cells from healthy controls [97]. They speculated that reduced production of IFNs may play a role in virus-induced COPD exacerbations. A second study reported a reduction in baseline mRNA expression of IFNβ as well as of IFNλ2/3, but not IFNλ1, in sputum cells from patients who experience frequent exacerbation compared to those who do not, while expression of all IFNs was reduced during virus-associated naturally occurring exacerbations [100]. In further support of this concept, García-Valero et al. reported reduced immunostaining and mRNA levels of IFNβ and interferon regulatory factor-7 (IRF-7) at baseline in COPD patients compared with healthy smokers and never-smokers [101].

**Figure 2:**
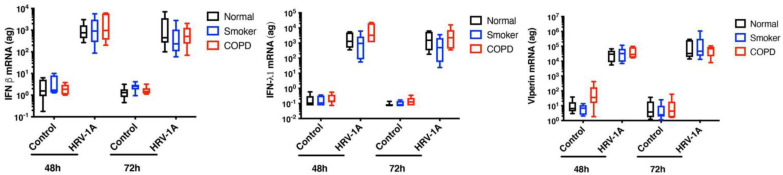
Exposure of bronchial epithelial cells from COPD patients, healthy smokers and non-smokers to rhinovirus-1A lead to similar levels of IFNβ, IFN-λ1 and viperin induction (*n* = 6). Bronchial epithelial cells were obtained by bronchial brushings. Cells were infected with rhinovirus for 48 h or 72 h. Levels of mRNA were assessed by real-time PCR.

The potential role of IFN deficiency is controversial, however, as other studies suggest no impairment of type I and type III IFN responses in COPD. Schneider and colleagues reported that airway epithelial cells isolated from patients with COPD and infected with rhinovirus showed increased rhinovirus replication compared to cells from healthy controls, despite increased interferon IFN-λ1 and IFN-λ2 levels in the COPD samples [102]. Baines and co-workers also observed increased expression of type I and type III IFNs in rhinovirus-infected epithelial cells from COPD patients compared to healthy controls but saw no difference in levels of rhinovirus replication between the two populations [103]. A study of sputum cells from COPD patients and healthy controls also found no differences in type I and type III IFNs between the two populations. In the population as a whole, data on ISGs were inconsistent, although a decrease was observed in the subgroup of patients with the most severe COPD [104]. Our own data also did not show any differences in levels of IFNβ and IFN-λ1, or of the antiviral protein viperin, upon rhinovirus infection of epithelial cells obtained from bronchial brushings from controls, healthy smokers and COPD patients (Figure 2). Interestingly, a recent study has proposed that any differences between COPD and healthy controls may be not due to a deficient airway epithelial IFN response but to one which is delayed, as they observed that maximal IFN production did not occur until significantly later in COPD patients [105].

Although a large number of antiviral genes are often referred to as ISGs because they can be induced by IFNs, these genes can also be induced independently of IFNs. Thus, Schmid and coworkers demonstrated that cells deficient in both type I and type III IFN receptors could still produce ISGs in response to viral infection [106]. Similarly, we have shown that cigarette smoke extract suppresses induction of numerous ISGs including viperin, ISG56 and ISG15 during rhinovirus infection in the absence of any significant changes in expression of type I or type III IFNs [107].

Evidence for a role of other respiratory viruses in exacerbations of COPD is more circumstantial. A review of multiple studies concluded that annual influenza vaccination is associated with reduced exacerbation and hospitalization in COPD [108]. Cigarette smoke exposure has been shown to reduce antiviral host defense in airway epithelial cells and to increase RSV replication [109,110]. RSV infections also exacerbate cigarette smoke-induced COPD in mice [111], and passive smoke exposure increases the severity of RSV induced airway disease in infants [112]. Substantially more work is needed, however, to definitively establish a direct mechanistic link between these viral infections and COPD exacerbations.

## 6. Viral–Bacterial Co-Infections

Viral–bacterial co-infections are detected in approximately 25% of patients hospitalized with exacerbations of COPD, and subjects with such co-infections were found to have more marked impairment of lung function and were hospitalized for longer [16]. It has also been observed that patients with viral–bacterial co-infections had more severe exacerbations and were at greater risk for readmission after their exacerbation [17].

Although it is conceivable that some of these co-infections occur via spontaneous independent viral and bacterial infections, there is clear evidence that viral infections can serve as a trigger for a secondary bacterial infection. Experimental rhinovirus infection of patients with moderate COPD, as well as smokers with normal lung function and healthy non-smokers resulted in secondary bacterial infections in 60% of patients with COPD but in only 9.5% of smokers with normal lung function and 10% of non-smokers [113]. The most common bacterial species observed in COPD patients were *Haemophilus influenzae* and *Streptococcus pneumoniae*. It is not clear, however, how many of these secondary bacterial infections represent de novo infections with new bacterial strains, since it has also been shown that experimental rhinovirus infection can lead to outgrowth of the bacterial airway microbiome and an increase in bacterial burden, with *Haemophilus influenzae* being the primary bacteria showing outgrowth [114]. Such changes in the microbiome were not observed in health individuals after rhinovirus infection.

**Figure 3:**
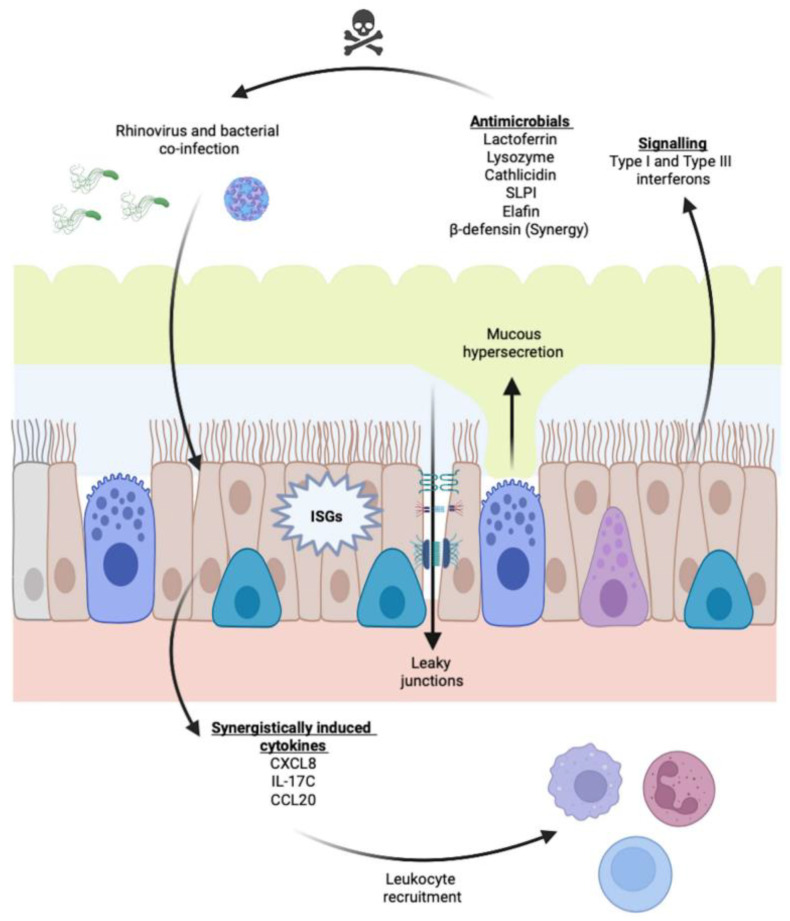
Airway epithelial responses to infection with viruses and/or bacteria. Epithelial cells produce intracellular and secreted antivirals and antibacterial products. In addition, chemokines and cytokines can be synergistically produced upon co-infection. There is also increased mucus secretion and a reduction in barrier function. ISGs, interferon-stimulated genes.

It is unclear what predisposes patients with COPD to secondary bacterial infections, although it has been suggested that observed reductions in levels of the antibacterial peptides secretory leukocyte protease inhibitor (SLPI) and elafin in COPD patients may contribute [113]. However, interactions of rhinovirus and bacteria with the airway epithelium are complex, leading to altered barrier function, as well as to production of proinflammatory cytokines and a host of antimicrobial molecules (Figure 3). Thus, other mechanisms may also contribute to bacterial infection and growth. It has been reported that rhinovirus infection can stimulate release of planktonic bacteria from biofilm and can increase inflammatory chemokine production from epithelial cells of patients with cystic fibrosis [115], but parallel studies have not been performed in cells from COPD patients. Rhinovirus has also been shown to upregulate airway epithelial expression of the bacterial adhesion molecules fibronectin, platelet-activating factor receptor (PAF-r) and carcinoembryonic antigen-related cell adhesion molecule (CEACAM), leading to increased adhesion of bacterial species including *Haemophilus influenzae, Staphylococcus aureus,* and *Streptococcus pneumoniae* [116], although it is not known if this occurs to a greater extent in cells from COPD patients. It is also well established that rhinovirus can disrupt epithelial barrier function [117,118,119], and such rhinovirus-induced disruption has been associated with increased paracellular migration of *Haemophilus influenzae* [117]. Finally, rhinovirus infections may impair some epithelial antibacterial responses to enhance bacterial growth. Infection with rhinovirus has been shown to promote degradation of the signaling molecule interleukin 1 receptor associated kinase 1 (IRAK1), leading to reduced epithelial production of CXCL8 and impaired neutrophil chemotaxis in response to non-typeable *Haemophilus influenzae* [120].

Once both viruses and bacteria are present in the airway, they can interact to modify epithelial responses relative to those observed with each stimulus individually. It has been shown that rhinovirus and bacteria synergistically induce epithelial production of several molecules, including CCL20, human β-defensin-2 (HBD2) and IL-17C [121,122,123]. These molecules are induced by synergistic effects on several signaling pathways. Interestingly, the antibacterial molecule HBD2 is produced in significantly lower amounts in epithelial cells from patients with COPD compared to those from smokers with normal lung function or from healthy controls [122]. By contrast, IL-17C, which feeds back on epithelial cells to stimulate production of the proinflammatory chemokine CXCL1, inducing neutrophil chemotaxis, is produced in increased amounts in response to virus and bacteria in cells from COPD patients compared to other groups [123]. It is tempting to speculate that this change in the balance to a reduced antimicrobial production but enhanced proinflammatory response in the airways may contribute to the combined effects of viral–bacterial co-infection on symptoms during COPD exacerbations.

## 7. Current Treatment of COPD Exacerbations

The current available pharmacological treatments for exacerbations of COPD have remained largely unchanged for some considerable time and rely on bronchodilators, corticosteroids and, when indicated, antibiotics. Despite the lack of strong evidence from randomized clinical trials, guidelines recommend that short-acting inhaled β_2_-adrenergic agonists, with or without short-acting anticholinergics be used as initial bronchodilators for treatment of acute exacerbations of COPD [1]. Although there are no clinical studies that have evaluated the use of long-acting bronchodilators during exacerbations, it is recommended that such treatments be continued during exacerbations.

In addition, to bronchodilator therapy, the use of oral corticosteroids is recommended as there is evidence that these drugs shorten recovery time, improve oxygenation, improve lung function and shorten hospitalization times [124,125,126,127]. It is recommended, however, that corticosteroid treatment be limited to 5 days to reduce the risk of development of pneumonia that has been observed with longer courses [128]. Although the mechanisms by which corticosteroids increase the risk of pneumonia are unclear, similar increases in risk have also been observed in patients receiving inhaled corticosteroids for significant durations [129,130].

As noted above, the use of antibiotics remains somewhat controversial because exacerbations may be triggered by viral as well as by bacterial infections, but they are considered beneficial in patients with purulent sputum as an indicator of a bacterial infection [87].

A major gap in our arsenal to treat exacerbations of COPD is any effective approach to treat virus-induced exacerbations. It has recently been shown that the monoclonal antibody, nirsevimab, given before the RSV season protected infants from medically attended RSV-associated lower respiratory tract infections [131], but it remains to be seen if this approach will be effective in patients with COPD. Moreover, while influenza vaccination is associated with a reduction in COPD exacerbations and hospitalization rates [108], this approach is not currently applicable to exacerbations triggered by other virus types. For example, no vaccine is available for use against rhinovirus infections. Development of such a vaccine is complicated by the existence of over 160 strains of rhinovirus that fall into three main groups (A, B and C) based on genome sequence homology [132,133]. This broad diversity, together with a lack of specific information on the critical, important immune-protective responses required to reduce symptoms, has led to a failure thus far to develop a vaccine to induce broad protective effects [134]. Similar to the challenges with vaccines, approaches to modulate broad-ranging immune response to rhinovirus infections have been unsuccessful. Although neutralizing antibodies to any given strain of rhinovirus are induced, these are usually highly strain specific [135], although RV-C species can induce some cross-species neutralizing responses [136]. It is also apparent that neutralizing antibodies do not develop until after peak symptoms have already occurred [137], raising concerns about protection during an ongoing exacerbation. Similar issues arise with cell-mediated immunity, which also tends not to develop until after symptoms begin to resolve. Although there is evidence of some shared T-cell epitopes among some species, this is neither consistent nor universal [138]. The delayed immune responses raise some questions about their role in viral clearance, a point also raised by the observation that highly differentiated airway epithelial cells in culture are able to clear rhinovirus infection in the absence of immune cells [139].

The other pharmacological approaches that have been used to try to limit rhinovirus infections have tried to prevent viral binding using either antireceptor antibodies or capsid-binding drugs or to prevent viral replication using drugs that inhibit viral proteases essential for replication [140,141]. Thus far, none of these attempts have led to successful therapies. So, we currently remain without any broad-ranging treatment for virus-induced exacerbations of COPD and additional approaches and further research are still needed.
